# A Coupled Biomechanical-Smoothed Particle Hydrodynamics Model for Horse Racing Tracks

**DOI:** 10.3389/fbioe.2022.766748

**Published:** 2022-02-21

**Authors:** Simon M. Harrison, R. Chris Whitton, Susan M. Stover, Jennifer E. Symons, Paul W. Cleary

**Affiliations:** ^1^ Data61, CSIRO, Clayton, VIC, Australia; ^2^ Faculty of Veterinary and Agricultural Sciences, University of Melbourne, Melbourne, VIC, Australia; ^3^ School of Veterinary Medicine, University of California, Davis, Davis, CA, United States; ^4^ University of Portland, Portland, OR, United States

**Keywords:** elastoplastic, biomechanics, equine, gait, quadruped, large deformation

## Abstract

Distal limb injuries are common in racing horses and track surface properties have been associated with injury risk. To better understand how track surfaces may contribute to equine limb injury, we developed the first 3D computational model of the equine hoof interacting with a racetrack and simulated interactions with model representations of 1) a dirt surface and 2) an all-weather synthetic track. First, a computational track model using the Smoothed Particle Hydrodynamics (SPH) method with a Drucker-Prager (D-P) elastoplastic material model was developed. It was validated against analytical models and published data and then calibrated using results of a custom track testing device applied to the two racetrack types. Second, a sensitivity analysis was performed to determine which model parameters contribute most significantly to the mechanical response of the track under impact-type loading. Third, the SPH track model was coupled to a biomechanical model of the horse forelimb and applied to hoof-track impact for a horse galloping on each track surface. We found that 1) the SPH track model was well validated and it could be calibrated to accurately represent impact loading of racetrack surfaces at two angles of impact; 2) the amount of harrowing applied to the track had the largest effect on impact loading, followed by elastic modulus and cohesion; 3) the model is able to accurately simulate hoof-ground interaction and enables study of the relationship between track surface parameters and the loading on horses’ distal forelimbs.

## Introduction

Forelimb injuries are common in racehorses often resulting in lameness and in severe cases death (Bailey et al., 1999; Parkin et al., 2006). The metacarpophalangeal (MCP) joint, or fetlock, is the site of most tendon, ligament, joint surface, and bone injuries (Bailey et al., 1999; Parkin et al., 2006). It is likely the MCP joint is prone to injury due to the large loads generated in this joint in galloping horses as a result of hyperextension during the stance phase of gait (Harrison et al., 2010, 2012, 2014).

Racetrack surface types have been associated with differences in musculoskeletal injury risk, with the likely reason being the effect of the surface on limb loading in the galloping horse. Typical track surfaces are dirt, sand, synthetic and turf. In North America turf and dirt tracks are associated with a higher risk of fatal and non-fatal fracture compared to synthetic tracks (Georgopoulos and Parkin 2017). In addition, track condition is associated with musculoskeletal injury rates with muddy dirt tracks and faster turf tracks having higher injury risk than fast dirt and slower turf tracks respectively (Hitchens et al., 2019).

Synthetic track material is typically a granular mix of sand, wax, and rubber particles. It has a different elastic and flow response to impact by the horse’s hooves than do dirt and sand tracks. Dirt and synthetic tracks are prepared with a loose surface layer the depth of which affects peak loads in drop tests simulating hoof strike and fetlock extension in galloping horses (Mahaffey et al., 2013; Symons et al., 2014). However, it is still unknown how the horse limbs are loaded during contact with dirt and synthetic track surfaces and how rheological differences affect the risk of injury during racing. Measurement of hoof-ground forces during racing is not practical so computational simulation is required to provide insight into the force transmission through the distal limb.

**FIGURE 1:**
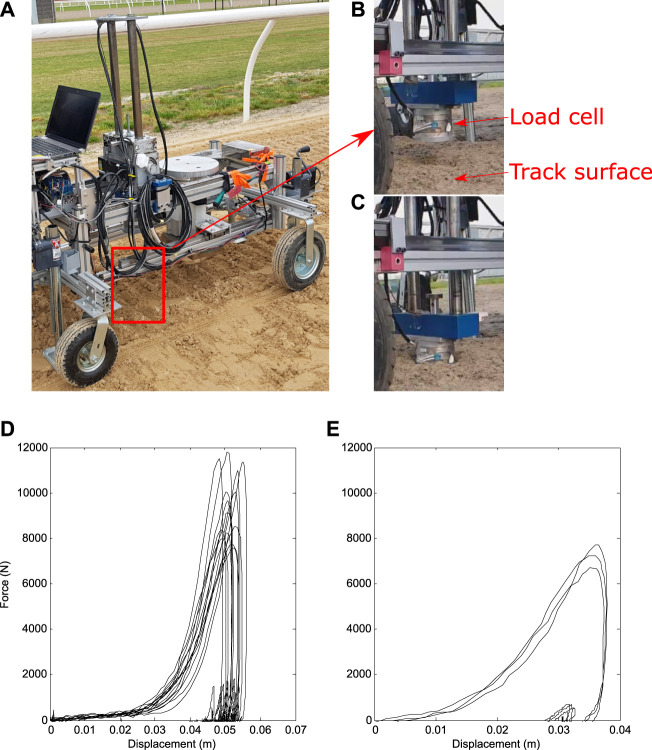
**(A–C)** Photographs of the track testing device (TTD) and ensemble force-displacement results for the TTD experiments as reported in (Setterbo et al., 2013) for **(D)** the dirt track and **(E)** synthetic (all weather) track. In the TTD experiments, a cylindrical mass is dropped onto a track from a known height. External force and distance travelled are recorded with the purpose of characterising the impact properties of the track.

Researchers at UCDavis have developed a track testing device (TTD) that measures the compressive and shear behaviour of a horse racetrack *in situ* (Setterbo et al., 2013). Figure 1 shows (a-c) photos of the TTD performing impact experiments and (d-e) example force-displacement results from Setterbo et al. (2013). Symons et al. (2015) used this device to calibrate a one-dimensional spring model of the track response that was successfully combined with a musculoskeletal model (Symons et al., 2016). It is not clear how the nonlinear spring model developed can be related to objective measures of stiffness, plasticity, friction angle and porosity of the track material and so there is motivation for a more detailed track surface model. In addition, Symons et al. (2016) reported deviations of model results from expectations when the hoof was substantially angled to the horizontal plane, all of which suggests that a three-dimensional model of track surfaces may be required to represent all hoof-track interactions accurately.

Computational modelling studies of equine distal limb loading during locomotion have been used to understand tendon and bone loading but there remain limits on their scope and application. Limitations include:

1. Use of gait speeds slower than those occurring during racing
2. The level of detail of the tendon, ligament, and muscle forces
3. The use of either very simple ground models or measured ground reaction data, or,
4. Focus on only the hoof and ignoring other body dynamics

Biewener (1998) developed the first comprehensive distal forelimb model, calculating some tendon forces that disagreed with experimental measures (Riemersma et al., 1996; Butcher et al., 2009) because distal joint torque constraints were ignored. Subsequent studies showed improved agreement with experimental measures by using distal joint torque constraints (Meershoek et al., 2001; Wilson et al., 2001; Swanstrom et al., 2005; Merritt et al., 2008), but contributions by separate elastic ligament structures were not predicted. Harrison et al. (2010) presented the most comprehensive and validated model to date, which included carpal muscle forces and more detailed representations of interactions between muscles, ligaments, and tendons. Only Reiser et al. (2000), Swanstrom et al. (2005) and Symons et al. (2016) have used forward dynamics to predict ground reaction force (GRF) and/or joint angles, but these models only considered 2D (sagittal plane) dynamics and used a simple spring model for the track that does not capture realistic elastic deformation and plastic flow behaviour.

Others have used models to predict the hoof-ground interaction without directly modelling the limb. Zhao et al. (2020) and Behnke (2017) used impact experiments and analytical (spring-dashpot) or 2D finite element (FE) models, respectively, to represent the hoof-ground interaction. They ignored the effects of joint movement during the hoof-ground contact, despite others showing the importance of these joint movements for moderating load (Wilson et al., 2001; McGuigan and Wilson, 2003). 3D models using Finite Element analysis (FEA) have been applied to hoof stresses in contact with different surfaces (treadmill, concrete, and sand) but the boundary conditions are typically generic or very simplistic and ignore the dynamics of the forelimb and torso (Newlyn et al., 1998; Hinterhofer et al., 2000; Hinterhofer et al., 2001; Thomason et al., 2002; Salo et al., 2010; Ramsey et al., 2013; Jansová et al., 2015; Akbari Shahkhosravi et al., 2021a; Akbari Shahkhosravi et al., 2021b). McCarty et al. (2016) presented the only study using FE to model both the response of the distal limb and the ground. They studied the dynamic impact of the hoof with the ground but did not consider muscle contractions or the full load cycle of the stance phase. A fully predictive model that incorporates all aspects of limb biomechanics and mechanical interactions with the track in three-dimensions is needed to better understand the relationship between gait movements, ground properties, internal body loading and injury.

Computer simulations of moving bodies interacting with flowing materials (such as water or soil in this study) require a mathematical method that can accurately represent large deformations and interactions with moving and deforming boundaries. A coupled biomechanical-Smoothed Particle Hydrodynamics (B-SPH) approach has been successfully used for human biomechanics in swimming (Cleary et al., 2013; Cohen et al., 2012, 2015, 2018), platform diving (Harrison et al., 2016b) and kayaking (Harrison et al., 2019). The B-SPH framework can be adjusted to include the flow of soil-like ground (Harrison and Cleary, 2013) and equine locomotion (Harrison et al., 2016a).

The purpose of this study is to develop a B-SPH model of equine locomotion to enable the study of the effects of changes to track material response on external body loading. We present coupled 3D dynamic models of the forelimb, the dirt and synthetic tracks, and the hoof-track interactions during galloping. The SPH track model is more realistic and general than that used in previous studies because it is based on a 3D elastic-plastic representation of material response under loading from the hoof. Others have used SPH to investigate and validate models of soil dynamics for application areas such as landslides (Bui et al., 2008; Wang et al., 2019; Zhan et al., 2020) or excavation (Li et al., 2018), but not biomechanics. The external and internal forces on the distal forelimb are calculated by combining the equine forelimb model given by Harrison et al. (2010) with a representation of the horse’s centre of mass and interactions with the SPH track surface. The model track properties were calibrated using the data from Setterbo et al. (2013) and the forelimb model was driven using 2D motion capture data on the same surfaces (Symons et al., 2014). The resulting model is systematically evaluated for use in predicting vertical impact force at the hoof, shown to be critical for understanding joint stresses (Harrison et al., 2014), and for understanding how track surface properties affect the magnitudes of these loads.

## Numerical Methods

The B-SPH model of hoof-track interaction is described in five stages:

1. A description of the SPH method and its application to impact/contact type scenarios
2. Verification of model resolution and validation of model outputs by comparison to analytical models and published simulation results
3. Calibration of track material parameters for the dirt and synthetic tracks described in Setterbo et al. (2013).
4. Sensitivity analysis of model outputs to changes in track material parameters
5. Demonstration of hoof-track interactions for cantering gait over the dirt and synthetic tracks

### The Smoothed Particle Hydrodynamics Method for Elastic Solids

SPH is a numerical method for solving partial differential equations (PDEs). It is a meshless Lagrangian method in which the governing equations are solved on a moving set of particles that represent discretised volumes of material. See Monaghan (1994) and Cleary (1998) for detailed explanations of the method and reviews by (Monaghan, 2005; Gómez-Gesteira et al., 2010). It has been used extensively to simulate the dynamics of elastoplastic solids (Chen et al., 2001; Gray et al., 2001; Cleary, 2010) and elastic-brittle fracture (Cleary and Das, 2008; Das and Cleary, 2008, 2010, 2013; Harrison and Cleary, 2014).

SPH is suited to solid mechanics applications where large deformations and/or damage occurs. Unlike more traditional methods such as Finite Volume and Finite Element analysis that solve for material motion using grids or meshes, SPH particles represent specific volumes of material and move with the material velocity. These particles carry information about physical properties of the system such as pressure, density, velocity, stresses, history dependent properties such as plastic strain and damage which is advected without numerical diffusion. Forces between particles are determined using a smoothing kernel function and are dependent on the distance between the particles. The use of the kernel function allows the governing partial differential equations (PDEs) of the physical system to be converted into spatially discretised systems of ordinary differential equations (ODEs), which can then be integrated forward in time to predict the state of the system.

The SPH continuity equation for fluids given by Monaghan (1994) in a form suitable for predicting elastic dynamics is:

$$\frac{d\rho_{a}}{dt}=\underset{b}{\sum }m_{b}v_{ab}\cdot \nabla_{a}W_{ab}$$ (1)

where *ρ*_*a*_ is the density of particle *a*, *t* is time, *m*_*b*_ is the mass of particle b, where ***v***_*ab*_ = ***v***_*a*_ - ***v***_*b*_ and **v**_a_ and **v**_b_ are the velocities of particles *a* and *b*. *W* is a cubic interpolation kernel function that is evaluated for the distance (magnitude of the vector **r**_ab_) between particles *a* and *b*. The kernel function and its properties are described in Monaghan (2005).

Conservation of momentum for elastic solids results in the following acceleration equation (Libersky and Petschek, 1991):

$$\frac{dv_{a}}{dt}=\underset{b}{\sum }m_{b}\left(\frac{\sigma_{a}}{\rho_{a}^{2}}+\frac{\sigma_{b}}{\rho_{b}^{2}}+\Pi_{ab}I\right)\cdot \nabla_{a}W_{ab}+g_{a}$$ (2)

where **σ**_a_ and **σ**_b_ are the stress tensors of particles *a* and *b*, respectively, *Π*_*ab*_ is an artificial representation of viscosity terms that result in both shear and bulk viscosity and ***I*** is the identity tensor. ***g***_*a*_ is the body force on particle *a* which in this case is gravity. The elastic stress tensor can be partitioned into a pressure part and a deviatoric stress component with deviatoric stress tensor, ***S***, and pressure *P*:

σ=−PI+S (3)

We use a linear model for the elastic stress versus strain relationship which gives a relationship between the pressure *P* and the density, ρ, typically referred to as an equation of state:

$$P=c^{2}\left(\rho -\rho_{0}\right)$$ (4)

where *ρ*_*0*_ is the reference density. The speed of sound *c* in the solid material is given by

$$c=\sqrt{\frac{K}{\rho_{0}}}$$ (5)

where *K* is the bulk modulus.

From Gray et al. (2001) the evolution of the deviatoric stress **S** is given in component form as:

$$\frac{dS^{ij}}{dt}=2G\left({\dot{\varepsilon }}^{ij}-\frac{1}{3}\delta^{ij}{\dot{\varepsilon }}^{kk}\right)+S^{ik}\Omega^{jk}+\Omega^{ik}S^{kj}\;\;\;$$ (6)

where $\dot{\varepsilon }$ is the strain tensor, $\delta^{ij}$ is the Kronecker delta, $\Omega^{jk}$ is the Jaumann rotation tensor, *G* is the shear modulus and indices *i*, *j* and *k* refer to three orthogonal directions in 3D space. The Einstein summation convention is used.

The strain rate tensor is calculated in an SPH form as

$${\dot{\varepsilon }}_{a}=-\frac{1}{2}\underset{b}{\sum }\frac{m_{b}}{\rho_{b}}\left[{\left(v_{ab}\nabla_{a}W_{ab}\right)}^{T}+v_{ab}\nabla_{a}W_{ab}\right]$$ (7)

and the Jaumann rotation tensor is expressed as:

$$\Omega_{a}=\frac{1}{2}\underset{b}{\sum }\frac{m_{b}}{\rho_{b}}\left[{\left(v_{ab}\nabla_{a}W_{ab}\right)}^{T}-v_{ab}\nabla_{a}W_{ab}\right].$$ (8)

The SPH method, particularly for elastic solids, can display tensile instabilities (Monaghan, 2000). The tensile instability correction proposed by Gray et al. (2001) is used here with a coefficient of 0.3 to inhibit these instabilities. This choice follows detailed evaluation of the tensile correction for SPH modelling of elastic solids in uniaxial compression tests (Das and Cleary, 2014).

### Smoothed Particle Hydrodynamics for Elastoplastic Dynamics

The results of the track tester experiments by Setterbo et al. (2013) show that both track materials demonstrate elastoplastic behaviour (see Figures 1D,E). A Drucker-Prager (D-P) model (Bui et al., 2008) is suitable for representing the dynamics of such elastoplastic materials. Details of the D-P model are given in Lemiale et al. (2012). The D-P model assumes the material to be initially elastic with a correction made to the pressure and deviatoric stress if any plastic deformation is predicted.

The D-P criterion for yielding is:

$$\tau -\alpha P\{\begin{array}{c} <k\;\text{if elastic}\\ \;\geq k\;\text{if yielding} \end{array}\;\;\;,$$ (9)

where *τ* is the shear yield stress and is given by:

$$\tau =\sqrt{\frac{1}{2}S^{ij}S^{ij}}$$ (10)

*α* is calculated from the friction angle, φ :

$$\alpha =\frac{6\sin\varphi }{\sqrt{3}\left(3-\sin\varphi \right)}$$ (11)

and *k* is the yield strength which is calculated from *α* and the cohesion, *c*.

$$k=\frac{6c\cos\varphi }{\sqrt{3}\left(3-\sin\varphi \right)}$$ (12)

The plastic deviatoric stress, ***S*** ^***P***^, is related to the elastic deviatoric stress, ***S***:

$$S^{P}=-\frac{G△\lambda }{\tau^{e}}S$$ (13)

The total deviatoric stress, S^T^, is a sum of elastic and plastic stresses

$$S^{T}=S+S^{P}=\left(1-\frac{G△\lambda }{\tau }\right)S$$ (14)

The plastic component of pressure, P^P^ is given by

$$P^{P}=K\beta △\lambda ,$$ (15)

where linear hardening is assumed and △λ is the increment of plastic strain,

$$△\lambda =\frac{\tau -\alpha P-k}{G+\alpha \beta K+\eta }\;\;\;\;,$$ (16)

*β* is calculated from *α* and the dilation angle ϕ

$$\beta =\frac{6\sin\phi }{\sqrt{3}\left(3-\sin\varphi \right)},$$ (17)

and *ɳ* is calculated from the hardening modulus *H*

$$\eta =\frac{6H\cos\varphi }{\sqrt{3}\left(3-\sin\varphi \right)}$$ (18)

The total pressure P^T^ is a sum of elastic and plastic components.

$$P^{T}=P+P^{P}$$ (19)

A second order predictor-corrector (explicit) integration scheme is used (see Monaghan, (2005) for details) with timestep, δt , chosen to be one-fifth of the Courant condition for stability of elastodynamic simulations:

$$\delta t=\frac{0.1h}{c}$$ (20)

where *h* is the SPH interpolation length.

### Interactions Between Smoothed Particle Hydrodynamics Particles and Boundaries

Solid boundaries are represented by triangular surface meshes. The nodes of the boundary mesh are represented in the SPH method as boundary particles with a penalty force applied in the normal direction. The force is calculated using a Lennard-Jones style form based on the orthogonal distance of the moving SPH particles from the solid surface (Monaghan, 1994). The penalty force replaces the pressure force terms in the momentum equation (Equation 2) for elastic-boundary particle pairs. Non-slip boundary conditions in the directions tangential to the solid surfaces are implemented by including the elastic-boundary SPH particle pairs in the summations for the artificial viscosity in Equation 2. For moving bodies, the nodal positions and the normal vectors are updated at each time-step to reflect the current position of the surface. This is a flexible boundary implementation that allows very complex solid boundaries (Cleary et al., 2006a; 2006b), moving boundaries (Cleary et al., 2007) and deforming boundaries (Cohen et al., 2012; Harrison et al., 2016b) to be modelled.

### Prior Validation of the Smoothed Particle Hydrodynamics Method for Solid Mechanics Applications

SPH has been shown to produce valid predictions for a large range of complicated behaviours of solid matter undergoing processes like those considered in the present work. Validation of the SPH method for these processes include comparison against exact solutions (Gray et al., 2001) and FEM solutions for uniaxial, biaxial, and loading of elastic solids (Das and Cleary, 2008, 2014; Pereira et al., 2017; Rausch et al., 2017), simple loading of beams and tensile failure under uniaxial loading (Ganzenmüller, 2015), and the deformation and failure of thin shelled materials (Maurel and Combescure, 2008). Other validations involve the comparison of simulation results with experimental data, for instance for fracturing of soft tissue (Rausch et al., 2017) and ice (Zhang et al., 2017), and machine cutting of metals (Limido et al., 2007; Xi et al., 2014).

### Prior Validation of the Smoothed Particle Hydrodynamics Code

It is not sufficient to rely on general validation of the SPH method. Additionally, it is necessary to validate the specific code implementation used. The implementation used in this study has been validated for simulations of elastic/elasto-brittle solids. Das and Cleary compared stress wave attributes calculated by the SPH code to those calculated using a commercial finite element (FE) code for uniaxial, biaxial and triaxial compression of an elastic object (Das et al., 2007; Das and Cleary, 2008, Das and Cleary, 2014). The SPH solutions were found to agree very well with analytical and FEM model solutions. SPH was shown to be stable and robust for elastodynamic applications, predicting a smoother response than the FEM code in the early stages of loading.

## Simulation Configurations

### Smoothed Particle Hydrodynamics Track Structure Models

The dirt and synthetic racetracks used by Setterbo et al. (2013) differed in their material type and geometric structure. The tracks were of different depths and below each track were hard substrate such as rock. Each track was harrowed prior to the measurement which causes the top region of the track to be aerated and therefore have a lower resistance to deformation. The dirt track had a depth of 0.5 m and was harrowed to a depth of 8.6 cm. The synthetic track was 0.26 m deep and was harrowed to a depth of 5.0 cm.

The developed models of each track were designed to specifically represent the track geometry, material behaviour and harrowing depth. The hard under-surface was modelled as a no-slip boundary. The aeration of the top section of the track by harrowing was modelled by randomly removing SPH particles from an initially densely packed array of particles to give a specific void space, which is the proportion of the volume that is air. The lower intact (non-harrowed) section remains solid, i.e. does not have any SPH particles removed. The geometric region modelled for the two track types are:

• For the dirt surface, a 0.5 m high x 1.0 m wide x 1.0 m long section of the track is discretised into 3.6 million SPH particles that are spaced at 5 mm. The harrowed region at the top is 8.6 cm deep.
• For the synthetic surface, a 0.26 m high x 1.0 m wide x 1.0 m long section of the track is discretised into 1.8 million SPH particles that are spaced at 5 mm. The harrowed region at the top is 5.0 cm deep.

**FIGURE 2:**
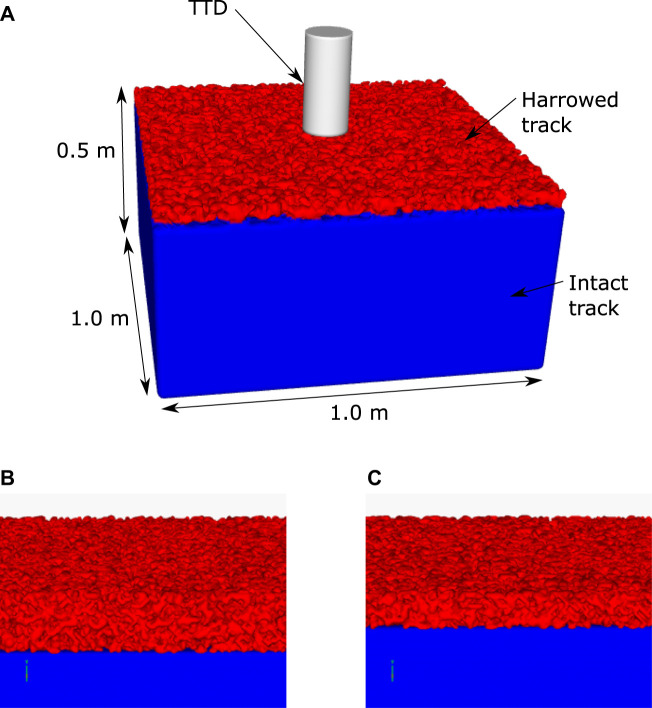
Configuration of the simulation configuration for the track testing device. The configuration for the dirt track is shown in **(A)**. The top layer of the track has a large number of voids in the material to represent harrowing that is used to break up and soften the track surface. The track extends 0.5 m in depth, below which the ground is predominantly rock. The rock is modelled as a rigid boundary condition. The synthetic track has a depth of 0.26 m under which a rigid boundary condition is also used. Close-up views of the dirt track and synthetic track models are shown in **(B)** and **(C)** respectively, which show the non-smooth top surface created by harrowing. The track testing device (TTD) is dropped and the force and displacement are predicted by the simulation. These results are compared to the matching experimental measurements in order to calibrate the rheological component of the model for the deformation of each track surface.

Figure 2 shows the standard configuration of the calibration simulations for the two track types.

### Rigid Body Model of the Track Testing Device

The TTD is a solid mass that is dropped onto the track surface during mechanical characterisation experiments (Setterbo et al., 2013). The device comprises a 27.8 kg, 12.7 cm diameter mass that, when dropped, travels down linear shafts until impact with the ground. It is represented in the model by a cylindrical mesh comprised of 1,700 nodes and 3,500 elements with average node spacing of 10 mm. The TTD is initially at rest with its lower surface at a height of 40.2 cm (Setterbo et al., 2013). The model TTD object is dynamically free to move in the vertical direction. External force on the TTD structure is calculated and the motion of the TTD in the vertical direction was predicted and recorded in each simulation.

### Setup for the Horse Biomechanical Model

**FIGURE 3:**
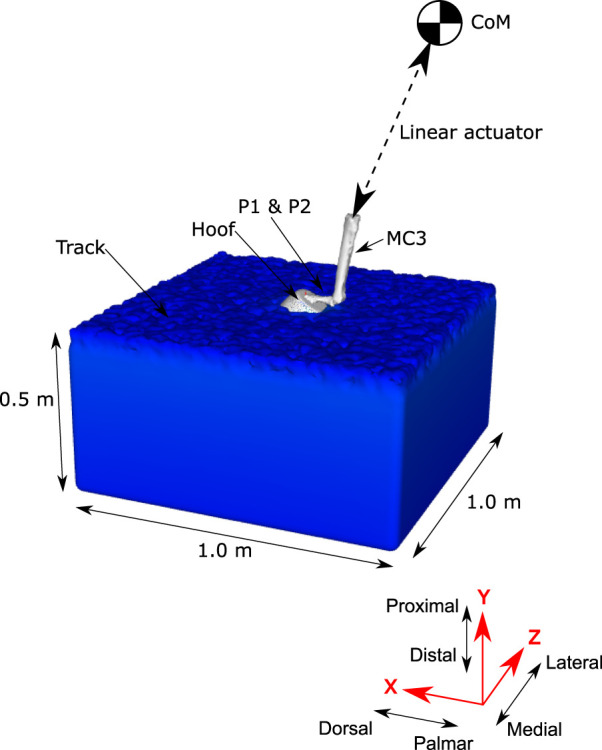
Schematic of the forelimb skeletal model used to simulate hoof-track forces during locomotion. The SPH track model is the same as used for *Calibration of the Track Model Material Properties Using Data From the Track Testing Experiments* section. The forelimb is represented by surface meshes of the distal bones (for visualisation purposes) and the outside surface of the hoof. The position and orientation of the hoof is prescribed from motion capture data, but the vertical position of the hoof is predicted by the simulation.

A model representation of one horse was developed using rigid body representations of the body limbs and a surface mesh representation of the hoof. Body kinematics were collected for the horse during a canter on the dirt and synthetic track surfaces (Symons et al., 2014). A generic geometric model of a hoof was developed from CT scans (850 node mesh and 1,700 triangular elements, average node spacing of 10 mm) (Harrison et al., 2014) and modified to include geometric representations of the shoes used during the experiments (Symons et al., 2014). Inertial effects from the limbs not in contact with the ground were assumed to be negligible and will be the subject of future investigations. A four-segment skeletal model was used to represent the dynamics of the body (see Figure 3). The vertical position of the centre of mass (CoM) was predicted by the simulation. The remaining translational and rotational degrees of freedom of the CoM and the rotation of the lower limb joints were prescribed from the kinematic data. The position and orientation of the hoof mesh was calculated at each time step from the skeletal model configuration.

## Evaluation of the Accuracy of Smoothed Particle Hydrodynamics for Solid Mechanics Applications

Here we specifically validate the SPH track model by first determining the resolution of the SPH representation of the track required for accurate predictions and then by comparison of simulation results to:

1. An analytical model for cylindrical indentation of an elastic object
2. An analytical model for indentation of a rigid wheel into a cohesive elastoplastic object with a negligible friction angle
3. A finite element (FE) model of indentation of a rigid wheel into an elastoplastic object with a large friction angle.

### Cylindrical indentation of an Elastic Object

According to Sneddon (1965), assuming quasi-static loading (i.e. negligible inertial and gravity effects), the gradient of the force-displacement curve for small displacements (<5% of the cylinder diameter) is:

$$s=\frac{2GD}{\left(1-\upsilon \right)},$$ (21)

where *s* is the gradient of the force-displacement curve, *D* is the diameter of the cylinder, *G* is the shear modulus of the track surface, and *υ* is the Poisson ratio of the track surface.

**FIGURE 4:**
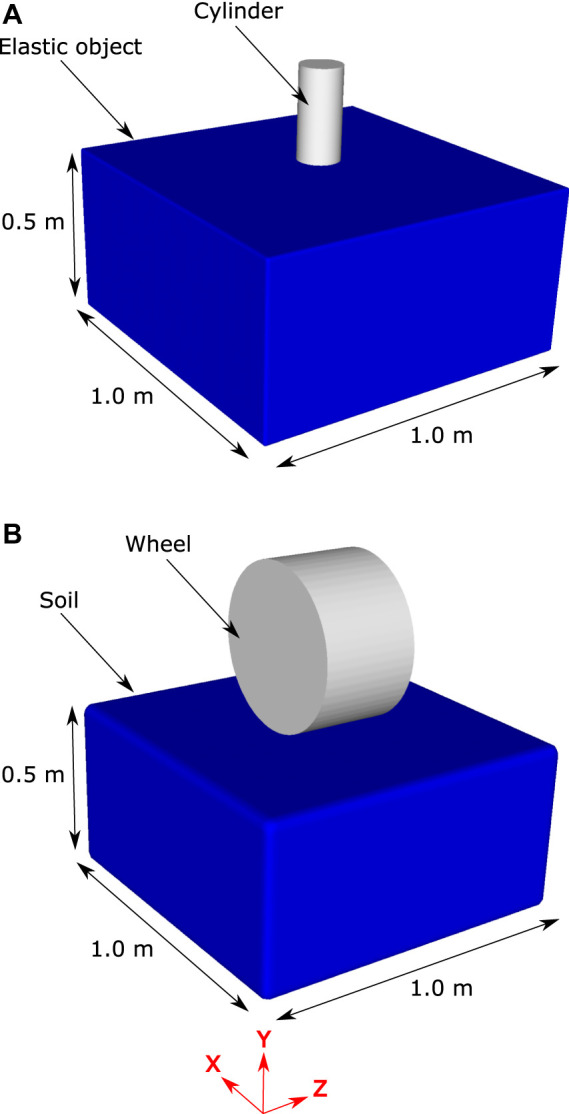
The two simulation configurations used in the verification and validation analysis. Panel **(A)** shows the configuration for cylindrical indentation of an elastic solid “soil”. Panel **(B)** shows the configuration for the indentation of an elastoplastic solid by a rigid wheel.

The simulation configuration is shown in Figure 4A. The elastic soil model is of dimensions 1.0 m × 0.26 m x 1.0 m and is represented by 1.8 million SPH particles that are spaced at 5 mm. All degrees of freedom of the SPH particles on all sides of the object except the top are fixed. The top surface of the elastic object is indented by a cylindrical shape in the form of a triangular mesh comprised of 1700 nodes and 3,500 elements. The indentor has a prescribed vertical velocity of 0.5 m/s and is initially positioned adjacent to the top surface of the elastic object. External force on the surface of the cylinder was recorded and the gradient of the simulated force-displacement results was compared to the analytical model for a range of SPH particle resolutions and three variations of elastic modulus.

**FIGURE 5:**
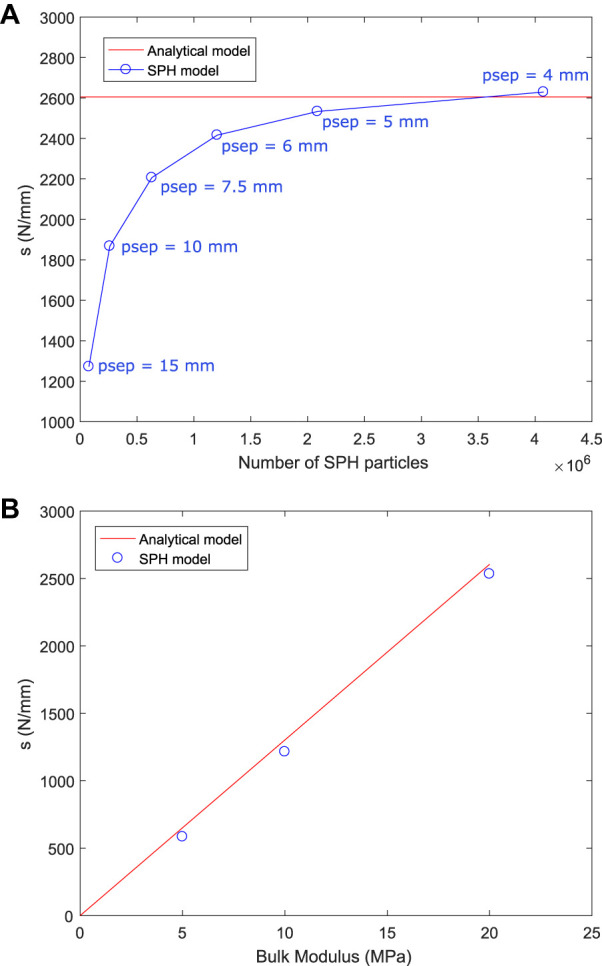
**(A)** Predicted slope of the force-displacement curve for the cylindrical indentation simulation for different SPH resolutions, compared to the analytical model result. Particle separations (psep) of 4–15 mm were used in separate simulations of the indentation problem. The simulation results can be considered converged in respect to particle size and verified against the analytical model for a particle size of 5 mm or smaller. **(B)** Predicted slope of the force-displacement curve for the cylindrical indentation simulation for different values of bulk modulus, compared to the analytical model result for an SPH resolution of 5 mm.

Simulation predictions of force and displacement were used to calculate the slope, *s*, in Eq. 21. Figure 5A shows the simulation prediction of *s* for SPH resolutions from 15 mm down to 4 mm. There are substantial differences in the results with range from 15 to 6 mm, but the results between 6 and 4 mm cases are very similar. There is a demonstrable convergence of results as particle size is decreased with the differences between the 5 and 4 mm case being sufficiently small (<5%) to justify the use of the 5 mm case for the remainder of the simulations. The smaller SPH particle size results converge to the value expected from the analytical model which verifies predictive behaviour of the SPH method and software for this configuration. Figure 5B shows the value of *s* predicted by the SPH model and by analytical model for three cases of bulk modulus for the cylindrical indentation simulation. There is very good agreement between the SPH and analytical models, confirming that the model is accurate across a wide range of elastic moduli.

### Indentation of a Rigid Wheel into Elastoplastic Soil

The SPH track model includes both elastic and plastic behaviour and the plastic model component must also be validated. Hambleton and Drescher (2008) presented an analytical model for a rigid wheel indenting soil with a negligible friction angle (which they termed a cohesive soil) and an FE model for a rigid wheel indenting soil with a large friction angle (which they termed a frictional soil).

Here we compare the results of the SPH track model to these two elastoplastic soil models. Figure 4B shows the SPH representation of the wheel indentation model published by (Hambleton and Dresher, 2008). A rigid wheel of diameter 0.5 m and thickness 0.3 m is positioned above a bed of SPH particles with dimensions: 0.5 m high x 1.0 m wide x 1.0 m long. The soil is represented by 150 thousand SPH particles that are spaced at 15 mm. The bulk and shear moduli of the soil are 4.1 and 1.9 MPa respectively. For the cohesive soil the cohesion is 6.1 kPa and the friction angle is 0°. For the frictional soil the cohesion is 61 Pa and the friction angle is 45°.

**FIGURE 6:**
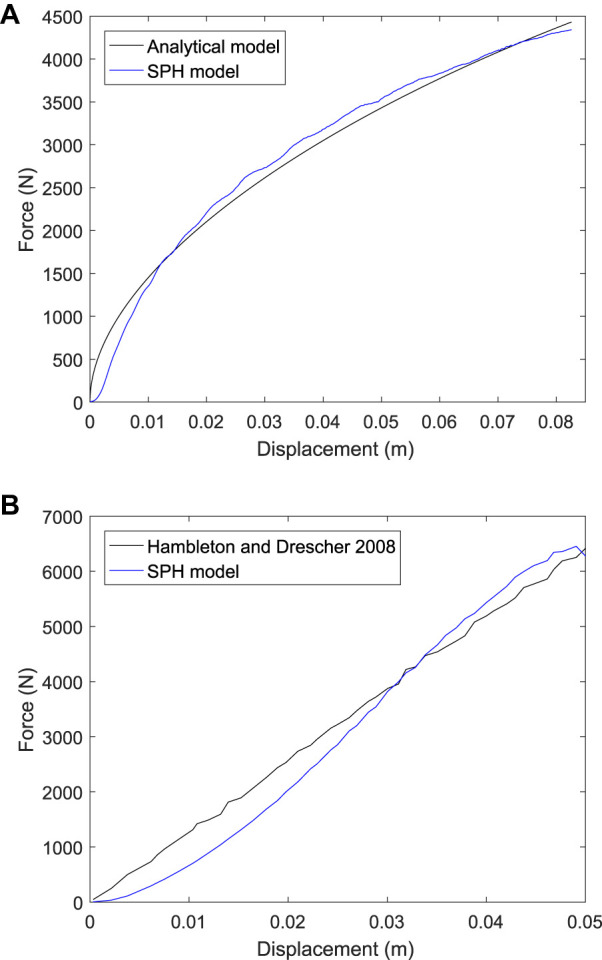
Force-displacement results from simulations of wheel indentation into **(A)** a cohesive soil, and **(B)** a frictional soil from (Hambleton and Drescher, 2008) and for the current SPH model.

Comparisons of the force-displacement results from the SPH model with those of (Hambleton and Dresher, 2008) are shown in Figure 6. Results for a cohesive soil are shown in Figure 6A. Very good agreement is observed with a root mean squared error (RMSE) of only 132 N, which is 3% of the maximum force. Results for a frictional soil are given in Figure 6B. Good agreement is seen between these and the FE model results of (Hambleton and Dresher, 2008). The RMSE is 390 N, which is an acceptable 6% of the maximum force. These results confirm that the SPH D-P model can sufficiently accurately predict the response of plasticity in soils of the type used in horse racing.

## Calibration of the Track Model Material Properties Using Data From the Track Testing Experiments

### Force-Displacement Data Measured by the Track Testing Device

Figures 1D,E shows the force-displacement results reported in Setterbo et al. (2013) for dirt and synthetic tracks. In each case the force trace rises slowly soon after contact and then rises sharply before peaking and dropping to zero quickly. The substantially different loading and unloading force traces indicate an elastoplastic response for both tracks. The force for the dirt track is less than for the synthetic track for small displacements but increases more sharply at large displacements resulting in a higher peak force. Despite the controlled nature of the impact there is a large amount of variability in data from repeats of the experiment, which suggests that the material is significantly inhomogeneous. During gait, the path and velocity of the hoof may vary significantly between strides and these effects combined suggest that stride-to-stride loading on the hoof could vary substantially.

### Calibration Method

SPH models of the dirt and synthetic tracks, as characterised by Setterbo et al. (2013), were constructed and calibrated. First, the geometry of each track was represented by specific simulation configurations (see *Smoothed Particle Hydrodynamics Track Structure Models*). Second, the elastic material properties of the track models were estimated from the TTD experiment results using an analytical model for cylindrical indentation (described in *Cylindrical indentation of an Elastic Object*). Third, the friction angle D-P material parameter was estimated using a characterisation of similar track materials from another study (Peterson et al., 2016). Fourth, TTD impact simulations were performed for each track surface for a vertical and 20° from vertical impact. Force-displacement results from the simulations were then compared to the measurements of Setterbo et al. (2013). The material parameters were iteratively adjusted to find an acceptable fit between model and experimental results.

### Deformation Behaviour

**FIGURE 7:**
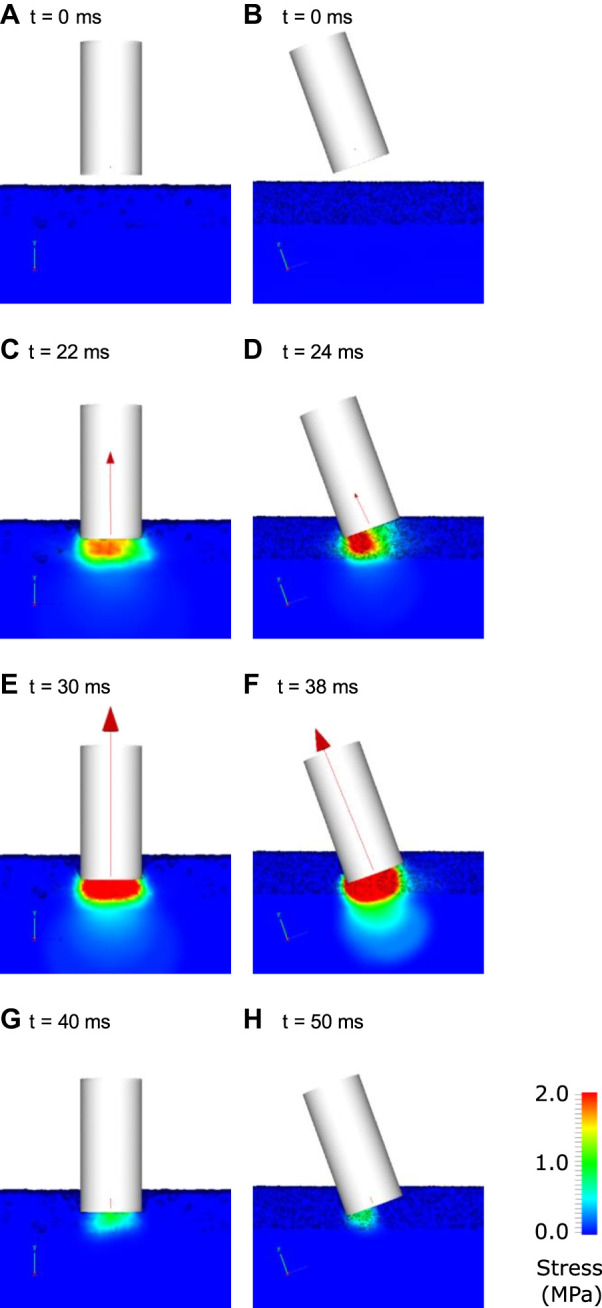
Visualisation of the interaction of the track testing device with ground for the case a dirt track. The left columns show the results for a vertical impact and the right column shows results for an angled impact. The track is coloured by von Mises stress at various times indicated by labels **(A–H)**. The net force on the TTD is shown as a red vector.

The TTD model material parameters were calibrated using the data from Setterbo et al. (2013) (Figures 1D,E). These material properties are listed in Table 1. Figure 7 shows deformation behaviour for the calibrated dirt track model in the virtual TTD test for a vertical impact (left columns) and an impact at 20° off-vertical alignment. For the vertical impact, the TTD contacted the track after 22 ms and a small vertical force is transmitted from the track surface into the TTD. Stresses in the harrowed section of the track directly below the TTD are substantial (>1 MPa) and the TTD compressed approximately half of the harrowed thickness of track. At 30 ms, the TTD has compressed the harrowed section of the track and as a result substantial stresses are induced in the non-harrowed section of the track below. At 40 ms, the TTD rebounds upwards and the stresses in the track and the force transmitted to the TTD decline.

**TABLE 1:** Calibrated elastic and D-P material parameters for the SPH model of each track material.

Material property	Dirt (harrowed)	Dirt (intact)	Synthetic (harrowed)	Synthetic (intact)
Depth of material (cm)	8.6	41.4	5	21
SPH resolution (mm)	5.0	5.0	5.0	5.0
Bulk modulus, *K* (MPa)	80	80	30	30
Shear modulus, G (MPa)	27	27	10	10
Cohesion, *c* (Pa)	2,500	2,500	250	250
Friction angle, φ (degrees)	45	45	45	45
Dilation angle, ϕ (degrees)	5	5	5	5
Harrowing (percentage material removed)	70%	0%	65%	0%

For the 20° off-vertical impact on the dirt track (Figure 7, right column), the stress under the TTD is higher under its lead side due to its greater penetration of the track (Figure 7D). The force is lower for the 20° angle impact (Figure 7D) than for the vertical impact (Figure 7C) at an equivalent of track penetration. This results in a smaller deceleration of the TTD for the angled impact compared to the vertical impact and therefore a later peak in force. The peak force occurs at 38 ms for the angled impact and the force is directed approximately through the centreline of the TTD. Between 38 and 50 ms, the TTD rebounds and stresses decay to zero.

**FIGURE 8:**
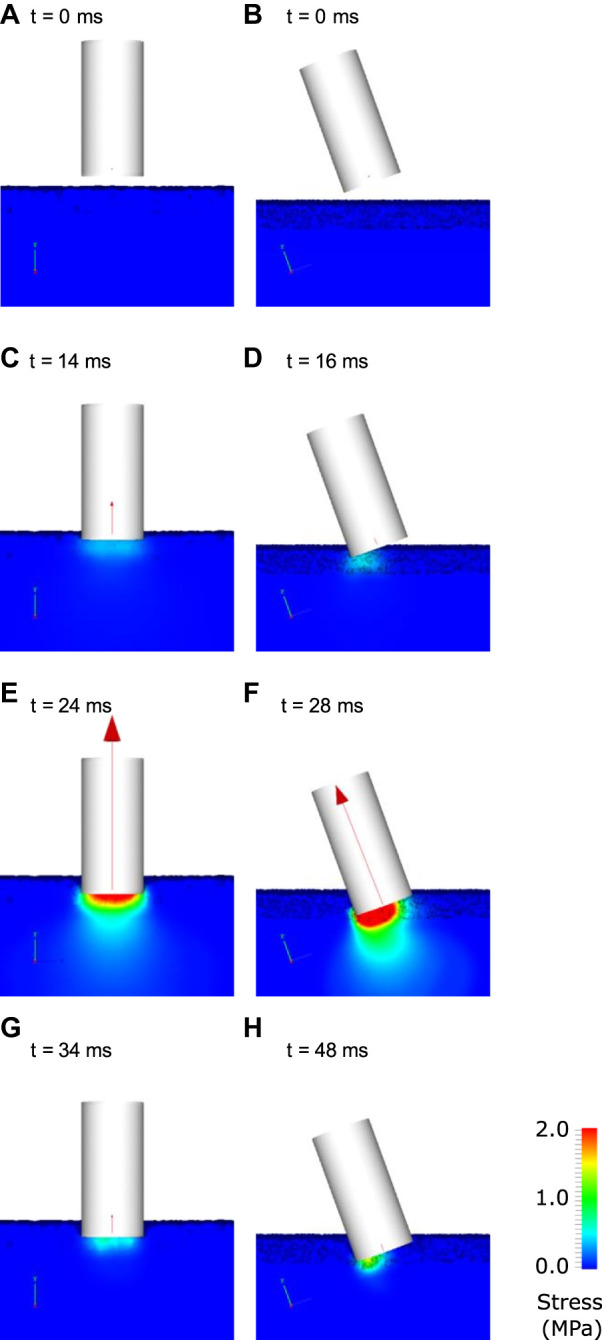
Visualisation of the interaction of the track testing device with ground for the case a synthetic track. The left columns show the results for a vertical impact and the right column shows results for an angled impact. The track is coloured by von Mises stress at various times indicated by labels **(A–H)**. The net force on the TTD is shown as a red vector.

Figure 8 shows deformation behaviour for the simulated TTD experiment for the calibrated synthetic track model. At 14 ms, the TTD contacted the track and a moderate vertical force is transmitted from the track onto the TTD. Stresses are less than 500 kPa and the deformation of the track is small. At 24 ms, the TTD has penetrated the track and substantial stresses have been induced in the track spreading radially from the TTD-ground contact surface. The peak force occurs at 24 ms, which is 6 ms earlier than for the dirt track. The stresses in the track are smaller than for the dirt track at maximum displacement. From 24 to 34 ms, the TTD rebounds upwards and the stresses in the track and the force imparted to the TTD decline. Similar to the dirt simulations, the angled impact produces higher stresses under the lead side of the TTD (Figures 8D,F,H). Peak force occurs 4 ms later than for the vertical impact due to the smaller contact area causing lower decelerating forces.

The complexity of the transient stress fields and the non-linear behaviour of the TTD motion highlight the strong need to include realistic predictions of the ground deformation and force response as opposed to using highly simplified spring-based interaction models.

### Calibrated Material Properties

**FIGURE 9:**
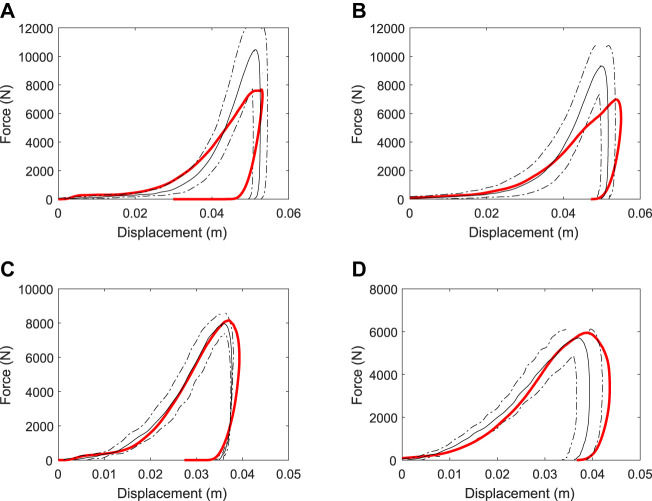
Variation of force-time results for simulations using the calibrated material parameters of the **(A,B)** dirt, and **(C,D)** synthetic tracks. Results for the vertical impact are shown in the left column (A, C) and for the 20° from vertical impact are shown in the right column (B, D). The experimental data is shown as mean (solid black line) ± standard deviation (dashed black lines) and the simulation data is shown as a red solid line.

Figure 9 shows the calibration curve for each track type for both the vertical and 20° from vertical impact experiments. Model predictions agreed well with the experimental results for both cases, especially for the initial loading response of the synthetic track and the peak force. This good agreement across different loading scenarios shows that a three-dimensional model can successfully represent the effects of different material properties, effects of harrowing (and the resulting void space in the upper region of track) and types of impacts without any changes to the underlying model. The unloading phase is moderately less well predicted by the model, suggesting that there is opportunity for further improvements such as taking account of the granular nature of some of the material and the rheological accuracy of the viscoelastic and/or plastic components of the model. Since peak loads are likely substantial contributors to injury, this level of accuracy is more than sufficient for the current purpose.

Table 1 lists the calibrated material parameters for the harrowed and non-harrowed (intact) sections for the SPH track models. The peak force transmitted to the TTD is higher for the dirt track than the synthetic track because it has a larger bulk modulus and cohesion. The softer initial response of the dirt track compared to the synthetic track occurs due to the higher void space of the harrowed track (70% void volume for the dirt track as compared to 65% for the synthetic track) and the larger depth of harrowing (8.6 cm for the dirt track as compared to 5 cm for the synthetic track). The dilation angle was found to have little effect on results and so the same values were used for both track surfaces.

## Sensitivity of Track Impact Response to Track Porosity and Elastoplastic Material Parameters

A first step towards using the track surface model for reducing racehorse injury is to understand the relationship between model results and variations in each model parameter. The D-P model has been used for simulating the mechanical response of soil during impact and landslides (Bui et al., 2008; Lemiale et al., 2012; López et al., 2012), but it has not previously been used to investigate loading on a body during exercise. Therefore, a sensitivity analysis was performed to determine the relative effect of each parameter on model results. The investigated material parameters included bulk modulus (*K*), cohesion (*c*), friction angle ( φ ), and the degree of porosity created by harrowing. Table 2 shows the ranges of the parameters investigated for each track type. In each simulation case the simulation parameter values were as described in Table 1, except for the one parameter being evaluated which was changed to the value indicated in Table 2. The outputs of each sensitivity analysis include the maximum displacement of the TTD, the gradient of the force-displacement curve during loading and the peak force.

**TABLE 2:** Variations in material parameters considered in sensitivity study.

	Dirt track			Synthetic track		
Parameter	Low	Baseline	High	Low	Baseline	High
Bulk modulus, *K* (MPa)	50	80	100	20	30	40
Cohesion, c (kPa)	0.25	2.5	25	0.025	0.25	2.5
Friction angle, φ (degrees)	25	45	65	35	45	55
Harrowing (percentage material removed)	60	70	80	60	65	70

**FIGURE 10:**
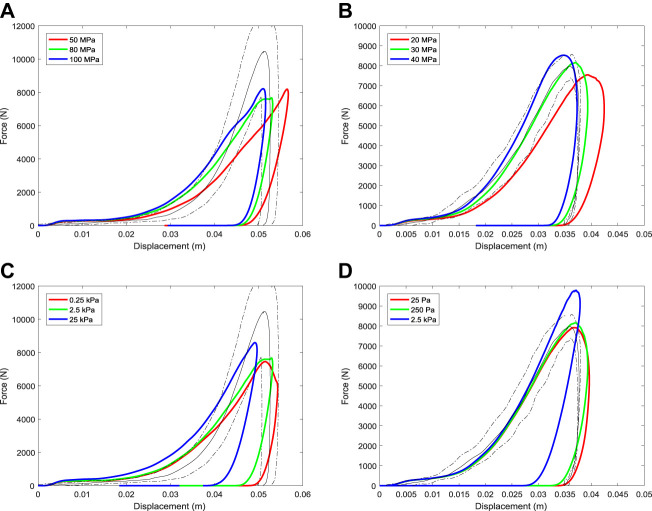
Variation of force-displacement results for the simulated track testing device with changes to **(A,B)** bulk modulus and **(C,D)** cohesion parameters of the track. Results for the dirt track are shown in the left column (a, c) for the synthetic track are shown in the right column (b, d).

Figure 10A and Figure 10B show the effect of changes to bulk modulus on the dynamic response of the two D-P track models. Increased bulk modulus increases the slope of the force trace for both the loading and unloading phases of the impact. The maximum displacement of the TTD is decreased for larger bulk modulus because force per unit displacement is increased leading to larger decelerating forces being imparted to the TTD. The peak force increases monotonically with increased bulk stiffness for the synthetic track, but not for the dirt track because the nonlinear effects of plastic flow (especially for the harrowed component) cause different force-displacement responses.

Figure 10C and Figure 10D show the effect of changes to cohesion parameter on the dynamic response of the D-P track models. An increase to cohesion substantially increases the force at initial impact and increases the peak load. The regions of track material below the TTD with stresses above the cohesion limit plastically flow away from the TTD, reducing the resistance to compression and therefore the load transmitted onto the TTD. Thus, maximum displacement is decreased when cohesion is increased. Increasing the load on the TTD leads to decreases in the duration of impact and the maximum displacement.

**FIGURE 11:**
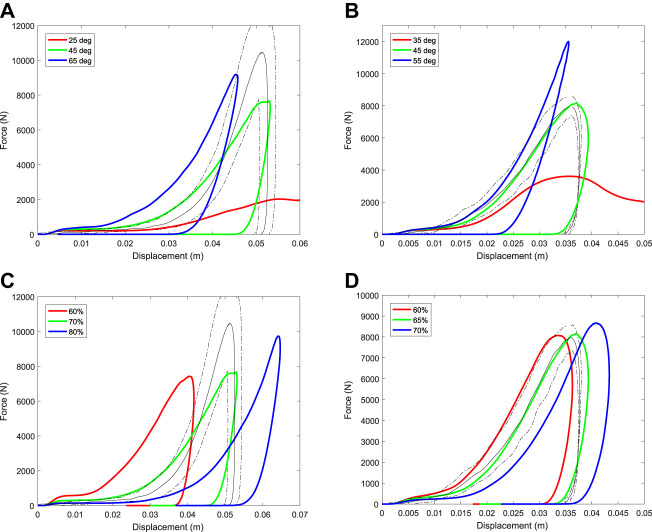
Variation of force-displacement results for the simulated track testing device with changes to **(A,B)** the friction angle parameter and **(C,D)** variations to the void space (or the volume proportion of air) in the harrowed upper section of track of the track. Results for the dirt track are shown in the left column (a, c) for the synthetic track are shown in the right column (b, d).

Force-displacement results are found to be sensitive to the friction angle (Figure 11A,B). Equations (9–12) show that friction angle controls the yield criteria and therefore affects whether the track responds to force with an elastic or plastic response. Increased friction angle increases the stress at which yielding occurs and as a result the track behaves elastically for longer and allows higher forces before yielding. The slope of the force-displacement curve (which is the effective stiffness of the track) is higher during loading with the peak displacement decreasing and peak force increasing (as should be expected).

The porosity of the top surface of the track following harrowing has the largest effect on the force-displacement results during impact (Figure 11C,D). Symons et al. (2016) also showed that harrowing has the highest influence on predicted force results using a 2D musculoskeletal model coupled to a 1D ground model. Porosity is quantified by a measure called void space, which is the proportion of the volume that is air. Increased void space in the top layer decreases the effective stiffness of the material and increases its ability to flow under load. As a result, an increase to the amount of modelled harrowing (and therefore void space) has a similar effect, simultaneously reducing both the bulk modulus and cohesion. Specifically, this substantially reduces the initial impact force and increases the maximum displacement and duration of impact. The penetration distance observed in experiment cannot be matched by the model if the void space is less than 70% for the dirt track (Figure 11C) and 60% for the synthetic track (Figure 11D). The void space of these harrowed tracks has not been measured, so it is not currently possible to evaluate these estimations of porosity.

## Application of the Biomechanics-Smoothed Particle Hydrodynamics Track Surface Model to Equine Locomotion

The motivation of the current work is to be able to simulate equine locomotion on racetrack surfaces to better understand the relationship between racetrack mechanical properties and loading at common injury sites in the distal forelimb. So as a final step we use the calibrated track surface model interacting with the horse limb biomechanical model to calculate loading on the fore-hoof for three cases of horses cantering on the same dirt and synthetic surfaces.

Simulations were run using openMP parallelisation over 36 cores and took 12 and 5.5 h for the dirt and synthetic surfaces, respectively. The difference in time taken for each simulation is directly attributable to the difference in bulk modulus between the two tracks models (Table 1), which determines the required timestep (Equation 20). The dirt material has a higher bulk modulus, therefore a smaller timestep, and thus more timesteps (and more computational time) to complete the full simulation.

**FIGURE 12:**
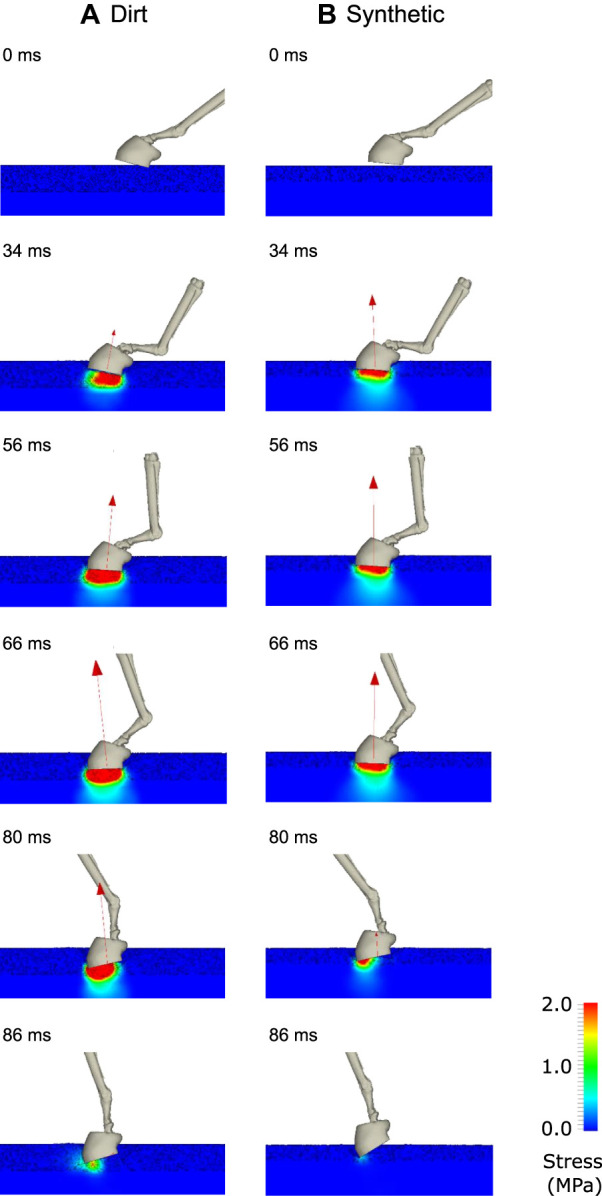
Visualisation of the gait simulations using the coupled B-SPH model for **(A)** the dirt track and **(B)** the synthetic track. The bones of the distal limb and the outside surface of the hoof are shown in each instant. The ground reaction force is shown as a red vector. The track surface is coloured by von Mises stress.

Visualisations of typical results for the coupled B-SPH track surface model results are shown in Figure 12. They are for one phase of the horse stance (the period in which one forelimb hoof is in contact with the ground). Initially, the hoof is above the ground and moving downwards. Stresses in the track are approximately zero (shown by the blue colour) since it is unloaded and in hydrostatic equilibrium.

At 34 ms, the downward moving hoof has made contact with the track surface. The harrowed track material (with visible voids) below the hoof experiences medium stress levels (light blue and green colours) as it compresses. The synthetic track (right) has a smaller harrowed depth (Table 1) and therefore (for a similar compressive displacement) has higher compressive stresses. The ground reaction force (GRF) vector is larger for the synthetic track than for the dirt track because of the higher stresses at this time in the stance phase. Yielding of the harrowed material occurs and the resulting plastic flow creates a footprint in the track. Negligible stress is transmitted to the non-harrowed material below the hoof because of the plastic flow of the intervening harrowed material. Between 56 and 80 ms, the stresses in the harrowed material increase in magnitude and the depth of the footprint increases. As the harrowed material below the hoof compresses, voids collapse leading to greater stress transmission, which allows stress to be transmitted through the non-harrowed basal material. The GRF increases substantially in magnitude once the harrowed material is largely compressed.

Since the dirt track has a larger depth of harrowing than the synthetic track, the increase in GRF occurs at a slower rate. Between 80 and 90 ms, the stance phase is completed and the hoof is lifted off the ground. This occurs earlier for the synthetic track than the dirt track because forces were imparted earlier (due to the lower amount of harrowing). As with the results of the previous section, the amount of harrowing appears to be the most significant factor contributing to the mechanical response of the hoof-track interaction.

**FIGURE 13:**
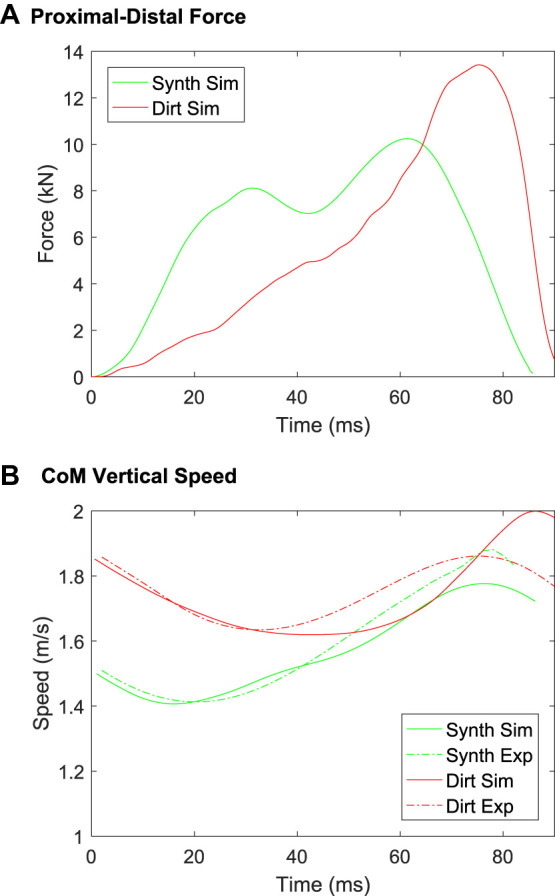
B-SPH model predictions of **(A)** ground reaction force and **(B)** centre of mass (CoM) speed in the proximal-distal (vertical) direction, for the synthetic and dirt track surfaces.

The model predictions of vertical ground reaction force and centre of mass speed are shown in Figure 13. The force for the synthetic track rises more quickly than for the dirt track, but peaks at a smaller force level than occurs for the dirt track (Figure 13A). The apparent softness of the dirt track early in the stance phase arises predominantly from the porosity of the structure and once compressed the material is less forgiving (from a stress transmission and peak force perspective for the horse). After the harrowed layer is sufficiently compressed and stresses are fully transmitted to the non-harrowed material, the force in the dirt track is higher than for the synthetic track. This occurs because the bulk and shear moduli are higher for the dirt track than for the synthetic track (Table 1). The large vertical forces predicted by the model will create very large flexion-extension torques about the distal joints, especially the fetlock joint, which are implicated in high joint stresses and elevated injury risk (Harrison et al., 2014). These model results show how hoof impact loading can be moderated by effective track surface design. Specifically, they show that changes in track elastic modulus and harrowing depth can affect both the loading rate and the peak amount of force imparted onto the hoof and therefore into the horse’s forelimbs. It is unclear however what type of track response is most suitable for reducing risk of musculoskeletal injury in the racehorse and this will be the focus of future applications of the musculoskeletal model.

Figure 13B shows a comparison of the vertical speed of the centre of mass as measured (from Symons et al., 2014) and as predicted by the B-SPH model. For both the dirt and synthetic tracks the measured and predicted speed during decelerating phase matches well (0–40 ms for the dirt track, 0–25 ms for the synthetic track). The agreement is moderately good for the synthetic track throughout the stance phase. The model predicts the upward movement of the centre of mass (40–80 ms) with lesser agreement for the dirt track. The results of Setterbo et al. (2013) show that there are large variations in force magnitude for the dirt track, especially for high levels of penetration depth. Considering the variation in the experimental results, we consider our predictions of ground reaction force and centre of mass speed to be sufficiently accurate for purpose.

Many aspects of these simulations can be compared to the work by others. Behnke (2018) used a 2D FE model to simulate the response of hoof-ground contact on asphalt and sand which was then used to calibrate a simple spring-dashpot model of the interaction. Limb dynamics (joint angle changes) were ignored. Their predictions for peak vertical forces were almost identical for the different surfaces. Symons et al. (2016) used a sequence of spring-dashpots and a dynamic limb model to evaluate the MCP joint angle on dirt and synthetic tracks. They predicted higher MCP joint angles, indicating high ground reaction forces (McGuigan and Wilson, 2003), for the dirt track than for the synthetic track. Our model predictions better agree with Symons et al. (2016) than the simpler model of Behnke, specifically that the peak vertical force is different for different surfaces (Figure 13A). Others have shown that SPH is effective for replicating soil behaviour due to its ability to deal with complex moving and deforming boundaries and the plastic flow of solid materials (Bui et al., 2008; Li et al., 2018; Wang et al., 2019).

## Future Model Extensions

Many aspects of the model will be extended for greater utility in understanding distal limb injury. Currently the model does not include the dynamics of all four limbs and this may contribute small amounts of error to both the predictions of ground reaction force and body dynamics. The model will be extended to include all body joints and their effects on body dynamics. Joint angles are prescribed from 2D kinematic measurements for specific track surfaces in the present study which precludes the model’s use for novel surface conditions. Joint angles will be predicted in future work from the load transmission between muscles, tendons, ligaments, and external forces. Stresses in the bones, cartilage, muscles, tendons, and ligaments can be predicted simultaneously by representing these structures using an SPH or FE approach, extending the ability for the modelling framework to investigate the cause of injury or disease in specific regions of the distal limb.

## Conclusion

A coupled B-SPH model of horse interaction with track surfaces is presented, which combines three-dimensional representations of the track (included the effects of harrowing), elastic and plastic deformation, the dynamics of the horse’s body, and the interactions between the track and the hoof. Being fully predictive, the model can be used to investigate the relationship between track surface properties and limb loading and may provide insight into the cause of common distal limb injuries.

The SPH track deformation model is validated by comparison to analytical models and finite element model results from the published literature. A particle resolution convergence analysis verifies that an SPH particle size of 5 mm is sufficient for accurate predictions of elastic and plastic dynamics. Validation analyses show that both the predicted elastic and plastic response of the track are sufficiently accurate.

The track model is calibrated for use with two different track surface types: dirt and synthetic (all weather). Previous experiments are reproduced *in silico* including the geometric structure of each track and the testing device. Elastic and plastic material parameters are determined by iteratively modifying them to produce an acceptable match between simulation predictions and experimental measures of force and displacement. The calibrated values of bulk and shear modulus are found to be higher for the dirt track than for the synthetic track. This difference in elastic properties is then identified as the cause of higher impact forces observed for the dirt track.

A sensitivity analysis is presented to demonstrate how such a predictive model can provide new insight into the way in which track modifications might reduce loading and presumably therefore injury risk. The analysis shows that the amount of track harrowing has a larger effect on loading during impact testing than any other factor. This result has been suggested by others with a different modelling framework (Symons et al., 2016). Variations in elastic modulus, cohesion and friction angle have a smaller, but still considerable, effect on model results and changes to dilation angle has a negligible effect on results.

Finally, the coupled B-SPH model is applied to equine locomotion over two track surfaces to demonstrate its use for investigating limb loading during racing-type gait. The rate of loading of the hoof during initial impact is higher for the synthetic track than for the dirt track, due to its lower porosity (or lesser harrowing). The peak force on the hoof is higher for the dirt track because the harrowed tracks are fully compressed by the hoof at the timing of each peak (thus eliminating the differences in harrowing between the tracks), and the elastic moduli and cohesion are both higher for the dirt track. Model predictions of vertical centre of mass speed are reasonable considering the variance of track material response. In future work, the model will be used to calculate tendon forces and joint stresses, and to predict gait responses to changes in track material properties so as to elucidate the relationship between track surface properties and injury risk.

## Data Availability

The datasets presented in this article are not readily available because of the very large size of simulation results files and the proprietary data format used. Requests to access the datasets should be directed to SH.

## References

[B1] Akbari Shahkhosravi N. C. R. Bellenzani M. M. S. Davies H. Komeili A. (2021a). The Influence of Equine Limb Conformation on the Biomechanical Responses of the Hoof: An *In Vivo* and Finite Element Study. J. Biomech. 128, 110715. 10.1016/j.jbiomech.2021.110715 34482223

[B2] Akbari Shahkhosravi N. Gohari S. Komeili A. Burvill C. Davies H. (2021b). Linear Elastic and Hyperelastic Studies of Equine Hoof Mechanical Response at Different Hydration Levels. J. Mech. Behav. Biomed. Mater. 121, 104622. 10.1016/j.jmbbm.2021.104622 34116431

[B3] Bailey C. J. Reid S. W. J. Hodgson D. R. Rose R. J. (1999). Impact of Injuries and Disease on a Cohort of Two- and Three-Year-Old Thoroughbreds in Training. Vet. Rec. 145, 487–493. 10.1136/vr.145.17.487 10596871

[B5] Behnke R. (2018). Numerical Time-Domain Modelling of Hoof-Ground Interaction during the Stance Phase. Equine Vet. J. 50, 519–524. 10.1111/evj.12782 29121424

[B6] Biewener A. A. (1998). Muscle-tendon Stresses and Elastic Energy Storage during Locomotion in the Horse. Comp. Biochem. Physiol. B: Biochem. Mol. Biol. 120, 73–87. 10.1016/s0305-0491(98)00024-8 9787779

[B7] Bui H. H. Fukagawa R. Sako K. Ohno S. (2008). Lagrangian Meshfree Particles Method (SPH) for Large Deformation and Failure Flows of Geomaterial Using Elastic-Plastic Soil Constitutive Model. Int. J. Numer. Anal. Meth. Geomech. 32, 1537–1570. 10.1002/nag.688

[B8] Butcher M. T. Hermanson J. W. Ducharme N. G. Mitchell L. M. Soderholm L. V. Bertram J. E. A. (2009). Contractile Behavior of the Forelimb Digital Flexors during Steady-State Locomotion in Horses (*Equus caballus*): an Initial Test of Muscle Architectural Hypotheses about *In Vivo* Function. Comp. Biochem. Physiol. A: Mol. Integr. Physiol. 152, 100–114. 10.1016/j.cbpa.2008.09.007 18835360

[B9] Chen J. K. Beraun J. E. Jih C. J. (2001). A Corrective Smoothed Particle Method for Transient Elastoplastic Dynamics. Comput. Mech. 27, 177–187. 10.1007/s004660100236

[B10] Cleary P. W. Cohen R. C. Z. Harrison S. M. Sinnott M. D. Prakash M. Mead S. (2013). Prediction of Industrial, Biophysical and Extreme Geophysical Flows Using Particle Methods. Eng. Computations 30, 157–196. 10.1108/02644401311304845

[B11] Cleary P. W. Das R. (2008). “The Potential for SPH Modelling of Solid Deformation and Fracture,” in IUTAM Symposium on Theoretical, Computational and Modelling Aspects of Inelastic Media (Springer), 287–296.

[B12] Cleary P. W. (2010). Elastoplastic Deformation during Projectile-wall Collision. Appl. Math. Model. 34, 266–283. 10.1016/j.apm.2009.04.004

[B13] Cleary P. W. Ha J. Prakash M. Nguyen T. (2006a). 3D SPH flow predictions and validation for high pressure die casting of automotive components. Appl. Math. Model. 30, 1406–1427. 10.1016/j.apm.2006.03.012

[B14] Cleary P. W. (1998). Modelling Confined Multi-Material Heat and Mass Flows Using SPH. Appl. Math. Model. 22, 981–993. 10.1016/s0307-904x(98)10031-8

[B15] Cleary P. W. Prakash M. Ha J. (2006b). Novel Applications of Smoothed Particle Hydrodynamics (SPH) in Metal Forming. J. Mater. Process. Techn. 177, 41–48. 10.1016/j.jmatprotec.2006.03.237

[B16] Cleary P. W. Prakash M. Ha J. Stokes N. Scott C. (2007). Smooth Particle Hydrodynamics: Status and Future Potential. Pcfd 7, 70–90. 10.1504/pcfd.2007.013000

[B17] Cohen R. C. Z. Cleary P. W. Mason B. R. Pease D. L. (2018). Forces during Front Crawl Swimming at Different Stroke Rates. Sports Eng. 21, 63–73. 10.1007/s12283-017-0246-x

[B18] Cohen R. C. Z. Cleary P. W. Mason B. R. Pease D. L. (2015). The Role of the Hand during Freestyle Swimming. J. Biomech. Eng. 137, 111007. 10.1115/1.4031586 26372433

[B19] Cohen R. C. Z. Cleary P. W. Mason B. R. (2012). Simulations of Dolphin Kick Swimming Using Smoothed Particle Hydrodynamics. Hum. Mov. Sci. 31, 604–619. 10.1016/j.humov.2011.06.008 21840077

[B20] Das R. Cleary P. W. (2013). A Mesh-free Approach for Fracture Modelling of Gravity Dams under Earthquake. Int. J. Fract. 179, 9–33. 10.1007/s10704-012-9766-3

[B21] Das R. Cleary P. W. (2010). Effect of Rock Shapes on Brittle Fracture Using Smoothed Particle Hydrodynamics. Theor. Appl. Fracture Mech. 53, 47–60. 10.1016/j.tafmec.2009.12.004

[B22] Das R. Cleary P. W. (2014). Evaluation of Accuracy and Stability of the Classical SPH Method under Uniaxial Compression. J. Sci. Comput. 64(3):858–897. 10.1007/s10915-014-9948-4

[B23] Das R. Cleary P. W. (2008). Modelling Stress Wave Propagation under Biaxial Loading Using SPH. in” Presented at the XXII International Congress of Theoretical and Applied Mechanics

[B24] Das R. Cleary P. W. others (2007). “Modelling Stress Wave Propagation and Triaxial Compression Test Using Smoothed Particle Hydrodynamics,” in Proceedings of the 5th Australasian Congress on Applied Mechanics (Brisbane, Australia: Engineers Australia), 659.

[B25] Ganzenmüller G. C. (2015). An Hourglass Control Algorithm for Lagrangian Smooth Particle Hydrodynamics. Comput. Methods Appl. Mech. Eng. 286, 87–106.

[B26] Gomez-Gesteira M. Rogers B. D. Dalrymple R. A. Crespo A. J. C. (2010). State-of-the-art of Classical SPH for Free-Surface Flows. J. Hydraulic Res. 48, 6–27. 10.1080/00221686.2010.9641242

[B27] Gray J. P. Monaghan J. J. Swift R. P. (2001). SPH Elastic Dynamics. Comput. Methods Appl. Mech. Eng. 190, 6641–6662. 10.1016/s0045-7825(01)00254-7

[B28] Hambleton J. P. Drescher A. (2008). Modeling Wheel-Induced Rutting in Soils: Indentation. J. Terramechanics 45, 201–211. 10.1016/j.jterra.2008.11.001

[B29] Harrison S. M. Chris Whitton R. Kawcak C. E. Stover S. M. Pandy M. G. (2014). Evaluation of a Subject-specific Finite-Element Model of the Equine Metacarpophalangeal Joint under Physiological Load. J. Biomech. 47, 65–73. 10.1016/j.jbiomech.2013.10.001 24210848

[B30] Harrison S. M. Cleary P. (2013). Dynamic Prediction of Body Dynamics during Walking on Soft Surfaces. in” Presented at the 19th Annual Scientific Meeting of the Australian & New Zealand Orthopaedic Research Society

[B31] Harrison S. M. Cleary P. W. Cohen R. C. Z. (2019). Dynamic Simulation of Flat Water Kayaking Using a Coupled Biomechanical-Smoothed Particle Hydrodynamics Model. Hum. Mov. Sci. 64, 252–273. 10.1016/j.humov.2019.02.003 30822692

[B32] Harrison S. M. Cleary P. W. Symons J. Stover S. Pandy M. (2016a). A Computational Model of Hoof-Ground Dynamic Interaction for Evaluating Muscle and Joint Reaction Forces during Equine Locomotion. Equine Vet. J. 48, 20.

[B33] Harrison S. M. Cleary P. W. (2014). Towards Modelling of Fluid Flow and Food Breakage by the Teeth in the Oral Cavity Using Smoothed Particle Hydrodynamics (SPH). Eur. Food Res. Technol. 238, 185–215. 10.1007/s00217-013-2077-8

[B34] Harrison S. M. Cohen R. C. Z. Cleary P. W. Barris S. Rose G. (2016b). A Coupled Biomechanical-Smoothed Particle Hydrodynamics Model for Predicting the Loading on the Body during Elite Platform Diving. Appl. Math. Model. 40, 3812–3831. 10.1016/j.apm.2015.11.009

[B35] Harrison S. M. Whitton R. C. Kawcak C. E. Stover S. M. Pandy M. G. (2010). Relationship between Muscle Forces, Joint Loading and Utilization of Elastic Strain Energy in Equine Locomotion. J. Exp. Biol. 213, 3998–4009. 10.1242/jeb.044545 21075941

[B36] Harrison S. M. Whitton R. C. King M. Haussler K. K. Kawcak C. E. Stover S. M. (2012). Forelimb Muscle Activity during Equine Locomotion. J. Exp. Biol. 215, 2980–2991. 10.1242/jeb.065441 22875767

[B37] Hinterhofer C. Stanek C. Haider H. (2001). Finite Element Analysis (FEA) as a Model to Predict Effects of Farriery on the Equine Hoof. Equine Vet. J. 33, 58–62. 10.1111/j.2042-3306.2001.tb05360.x 11721570

[B38] Hinterhofer C. Stanek C. Haider H. (2000). The Effect of Flat Horseshoes, Raised Heels and Lowered Heels on the Biomechanics of the Equine Hoof Assessed by Finite Element Analysis (FEA). J. Vet. Med. Ser. A 47, 73–82. 10.1046/j.1439-0442.2000.00263.x 10803106

[B39] Hitchens P. L. Morrice-West A. V. Stevenson M. A. Whitton R. C. (2019). Meta-analysis of Risk Factors for Racehorse Catastrophic Musculoskeletal Injury in Flat Racing. Vet. J. 245, 29–40. 10.1016/j.tvjl.2018.11.014 30819423

[B41] Jansová M. Ondoková L. Vychytil J. Kochová P. Witter K. Tonar Z. (2015). A Finite Element Model of an Equine Hoof. J. Equine Vet. Sci. 35, 60–69.

[B42] Lemiale V. Mead S. Cleary P. (2012). Numerical Modelling of Landslide Events Using a Combination of Continuum and Discrete Methods. in” Ninth International Conference on Computational Fluid Dynamics in the Minerals and Process Industries. Melbourne, Australia.

[B43] Li S. Chen X. Chen W. Zhu S. Li Y. Yang L. (2018). Soil-cutting Simulation and Parameter Optimization of Handheld Tiller's Rotary Blade by Smoothed Particle Hydrodynamics Modelling and Taguchi Method. J. Clean. Prod. 179, 55–62. 10.1016/j.jclepro.2017.12.228

[B44] Libersky L. D. Petschek A. G. (1991). “Smooth Particle Hydrodynamics with Strength of Materials,” in Advances in the Free-Lagrange Method Including Contributions on Adaptive Gridding and the Smooth Particle Hydrodynamics Method (Springer), 248–257.

[B45] Limido J. Espinosa C. Salaün M. Lacome J. L. (2007). SPH Method Applied to High Speed Cutting Modelling. Int. J. Mech. Sci. 49, 898–908. 10.1016/j.ijmecsci.2006.11.005

[B46] López Y. R. Roose D. Morfa C. R. (2012). Dynamic Particle Refinement in SPH: Application to Free Surface Flow and Non-cohesive Soil Simulations. Comput. Mech. 51, 731–741.

[B47] Mahaffey C. A. Peterson M. L. Roepstorff L. (2013). The Effects of Varying Cushion Depth on Dynamic Loading in Shallow Sand Thoroughbred Horse Dirt Racetracks. Biosyst. Eng. 114, 178–186. 10.1016/j.biosystemseng.2012.12.004

[B48] Maurel B. Combescure A. (2008). An SPH Shell Formulation for Plasticity and Fracture Analysis in Explicit Dynamics. Int. J. Numer. Meth. Engng 76, 949–971. 10.1002/nme.2316

[B49] McCarty C. A. Thomason J. J. Gordon K. D. Burkhart T. A. Milner J. S. Holdsworth D. W. (2016). Finite-Element Analysis of Bone Stresses on Primary Impact in a Large-Animal Model: The Distal End of the Equine Third Metacarpal. PLOS ONE 11, e0159541. 10.1371/journal.pone.0159541 27459189 PMC4961423

[B50] McGuigan M. P. Wilson A. M. (2003). The Effect of Gait and Digital Flexor Muscle Activation on Limb Compliance in the Forelimb of the horseEquus Caballus. J. Exp. Biol. 206, 1325–1336. 10.1242/jeb.00254 12624168

[B51] Meershoek L. S. Bogert A. J. v. d. Schamhardt H. C. (2001). Model Formulation and Determination of *In Vitro* Parameters of a Noninvasive Method to Calculate Flexor Tendon Forces in the Equine Forelimb. Am. J. Vet. Res. 62, 1585–1593. 10.2460/ajvr.2001.62.1585 11592324

[B54] Merritt J. S. Davies H. Burvill C. Pandy M. G. (2008). Influence of Muscle-Tendon Wrapping on Calculations of Joint Reaction Forces in the Equine Distal Forelimb. Biomed. Res. Int. 2008, 1–9. 10.1155/2008/165730 PMC2396215 18509485

[B55] Monaghan J. J. (1994). Simulating Free Surface Flows with SPH. J. Comput. Phys. 110, 399–406. 10.1006/jcph.1994.1034

[B56] Monaghan J. J. (2005). Smoothed Particle Hydrodynamics. Rep. Prog. Phys. 68, 1703–1759. 10.1088/0034-4885/68/8/r01

[B57] Monaghan J. J. (2000). SPH without a Tensile Instability. J. Comput. Phys. 159, 290–311. 10.1006/jcph.2000.6439

[B58] Newlyn H. A. Collins S. N. Cope B. C. Hopegood L. Latham R. J. Reilly J. D. (1998). Finite Element Analysis of Static Loading in Donkey Hoof wall. Equine Vet. J. Suppl. 30, 103–110. 10.1111/j.2042-3306.1998.tb05128.x 9932100

[B59] Parkin T. D. H. Clegg P. D. French N. P. Proudman C. J. Riggs C. M. Singer E. R. (2006). Catastrophic Fracture of the Lateral Condyle of the Third Metacarpus/metatarsus in UK Racehorses - Fracture Descriptions and Pre-existing Pathology. Vet. J. 171, 157–165. 10.1016/j.tvjl.2004.10.009 16427592

[B60] Pereira G. G. Cleary P. W. Lemiale V. (2017). SPH Method Applied to Compression of Solid Materials for a Variety of Loading Conditions. Appl. Math. Model. 44, 72–90. 10.1016/j.apm.2016.12.009

[B61] Peterson M. Vel S. Jin Z. (2016). Constitutive Modelling of Equestrian Surface Materials. Equine Vet. J. 48, 12.

[B62] Ramsey G. D. Hunter P. J. Nash M. P. (2013). The Influence of Loading Conditions on Equine Hoof Capsule Deflections and Stored Energy Assessed by Finite Element Analysis. Biosyst. Eng. 115, 283–290. 10.1016/j.biosystemseng.2013.04.002

[B63] Rausch M. K. Karniadakis G. E. Humphrey J. D. (2017). Modeling Soft Tissue Damage and Failure Using a Combined Particle/Continuum Approach. Biomech. Model. Mechanobiol. 16, 249–261. 10.1007/s10237-016-0814-1 27538848 PMC5288267

[B64] Riemersma D. J. Bogert A. J. Jansen M. O. Schamhardt H. C. (1996). Tendon Strain in the Forelimbs as a Function of Gait and Ground Characteristics and *In Vitro* Limb Loading in Ponies. Equine Vet. J. 28, 133–138. 10.1111/j.2042-3306.1996.tb01605.x 8706645

[B65] Salo Z. Thomason J. J. Runciman R. J. (2010). Analysis of Strain and Stress in the Equine Hoof Using Finite Element Analysis: Comparison with Minimum Principal Strains Recorded *In Vivo* . Biosyst. Eng. 107, 262–270. 10.1016/j.biosystemseng.2010.08.010

[B66] Setterbo J. J. Fyhrie P. B. Hubbard M. Upadhyaya S. K. Stover S. M. (2013). Dynamic Properties of a Dirt and a Synthetic Equine Racetrack Surface Measured by a Track-Testing Device. Equine Vet. J. 45, 25–30. 10.1111/j.2042-3306.2012.00582.x 22587378

[B67] Sneddon I. N. (1965). The Relation between Load and Penetration in the Axisymmetric Boussinesq Problem for a Punch of Arbitrary Profile. Int. J. Eng. Sci. 3, 47–57. 10.1016/0020-7225(65)90019-4

[B68] Swanstrom M. D. Zarucco L. Hubbard M. Stover S. M. Hawkins D. A. (2005). Musculoskeletal Modeling and Dynamic Simulation of the Thoroughbred Equine Forelimb during Stance Phase of the Gallop. J. Biomech. Eng. 127, 318–328. 10.1115/1.1865196 15971710

[B69] Symons J. E. Fyhrie D. P. Hawkins D. A. Upadhyaya S. K. Stover S. M. (2015). Modeling Equine Race Surface Vertical Mechanical Behaviors in a Musculoskeletal Modeling Environment. J. Biomech. 48 (4), 566–572. 10.1016/j.jbiomech.2015.01.006 25634662

[B70] Symons J. E. Garcia T. C. Stover S. M. (2014). Distal Hindlimb Kinematics of Galloping Thoroughbred Racehorses on Dirt and Synthetic Racetrack Surfaces. Equine Vet. J. 46, 227–232. 10.1111/evj.12113 23742040

[B71] Symons J. E. Hawkins D. A. Fyhrie D. P. Upadhyaya S. K. Stover S. M. (2016). Hitting the Ground Running: Evaluating an Integrated Racehorse Limb and Race Surface Computational Model. J. Biomech. 49, 1711–1717. 10.1016/j.jbiomech.2016.03.057 27086114

[B72] Thomason J. J. McClinchey H. L. Jofriet J. C. (2002). Analysis of Strain and Stress in the Equine Hoof Capsule Using Finite Element Methods: Comparison with Principal Strains Recorded *In Vivo* . Equine Vet. J. 34, 719–725. 10.2746/042516402776250388 12455844

[B73] Wang Y. Qin Z. Liu X. Li L. (2019). Probabilistic Analysis of post-failure Behavior of Soil Slopes Using Random Smoothed Particle Hydrodynamics. Eng. Geology. 261, 105266. 10.1016/j.enggeo.2019.105266

[B74] Wilson A. M. McGuigan M. P. Su A. van den Bogert A. J. (2001). Horses Damp the spring in Their Step. Nature 414, 895–899. 10.1038/414895a 11780059

[B75] Xi Y. Bermingham M. Wang G. Dargusch M. (2014). SPH/FE Modeling of Cutting Force and Chip Formation during Thermally Assisted Machining of Ti6Al4V alloy. Comput. Mater. Sci. 84, 188–197. 10.1016/j.commatsci.2013.12.018

[B76] Zhan L. Peng C. Zhang B. Wu W. (2020). A SPH Framework for Dynamic Interaction between Soil and Rigid Body System with Hybrid Contact Method. Int. J. Numer. Anal. Methods Geomech 44, 1446–1471. 10.1002/nag.3070

[B77] Zhang N. Zheng X. Ma Q. (2017). Updated Smoothed Particle Hydrodynamics for Simulating Bending and Compression Failure Progress of Ice. Water 9, 882. 10.3390/w9110882

